# Comparison of Eclipse Smart Segmentation and MIM Atlas Segment for liver delineation for yttrium‐90 selective internal radiation therapy

**DOI:** 10.1002/acm2.13668

**Published:** 2022-06-15

**Authors:** Jun Li, Rani Anne

**Affiliations:** ^1^ Department of Radiation Oncology Thomas Jefferson University Philadelphia Pennsylvania USA

**Keywords:** auto‐segmentation, liver delineation, Eclipse, MIM, resin yttrium‐90

## Abstract

**Purpose:**

The aim was to compare Smart Segmentation of Eclipse treatment planning system and Atlas Segment of MIM software for liver delineation for resin yttrium‐90 (Y‐90) procedures.

**Materials and methods:**

CT images of 20 patients treated with resin Y‐90 selective internal radiation therapy (SIRT) were tested. Liver contours generated with Smart Segmentation and Atlas Segment were compared with physician manually delineated contours. Dice similarity coefficient (DSC), mean distance to agreement (MDA), and ratio of volume (RV) were calculated. The contours were evaluated with activity calculations and ratio of activity (RA) was calculated.

**Results:**

Mean DSCs were 0.77 and 0.83, MDAs were 0.88 and 0.71 cm, mean RVs were 0.95 and 1.02, and mean RAs were 1.00 and 1.00, for Eclipse and MIM results, respectively.

**Conclusion:**

MIM outperformed Eclipse in both DSC and MDA, whereas the differences in liver volumes and calculated activities were statistically insignificant between the Eclipse and MIM results. Both auto‐segmentation tools can be used to generate initial liver contours for resin Y‐90 SIRT, which need to be reviewed and edited by physicians.

## 1 INTRODUCTION

Yttrium‐90 (Y‐90) selective internal radiation therapy (SIRT) is a promising procedure for liver cancer treatment.^1^ In a resin‐based Y‐90 SIRT procedure where the body‐surface‐area (BSA) method is used, tumor volumes and liver volumes are needed to calculate tumor involvement to determine Y‐90 activity.^2^ To obtain the volumes, physicians need to delineate the contours in 3D images (e.g., CT or MR images). In urgent cases, a quick turnaround of activity calculation is needed, which requires a quick contour delineation. It is desired to apply an auto‐segmentation tool in resin Y‐90 SIRT for liver delineation to expedite the activity calculation process.

In recent years, auto‐segmentation has been investigated for target and organ delineations in radiation therapy of various sites (prostate, head and neck, pelvis, and brain), with commercial software and researcher‐developed methods.^3^, ^4^, ^5^, ^6^, ^7^, ^8^, ^9^ For liver delineation, for instance, Yan et al. developed an atlas‐based method for applications using MR images.^10^ Lu et al. developed a graph cut–based method for CT images.^11^ A deep learning–based method was applied by Bousabarah et al. for liver segmentation in MR images.^12^

Varian Eclipse treatment planning system (Varian, Palo Alto, CA, USA) and MIM Maestro (MIM Software Inc., Cleveland, OH, USA) are two commercially available software popularly used in radiation therapy. Both systems provide auto‐segmentation tools. The aim of the study was to evaluate these two auto‐segmentation tools for potential applications in liver delineation for resin Y‐90 SIRT. Knowledge obtained in the study may be helpful to the applications in SIRT and other radiation therapy procedures.

## 2 METHODS

Liver auto‐segmentation performed with Varian Eclipse (version 15.6) and MIM Maestro (version 6.67) was evaluated. The auto‐segmentation tools are named Smart Segmentation in Eclipse and Atlas Segment in MIM Maestro, respectively. Both tools use atlas‐based segmentation methods. In this retrospective study, CT images of 42 patients who were treated with resin Y‐90 in our institution in recent years were included. The patients were randomly selected. Table 1 lists patient characteristics. Among them, CT images of 22 patients were used to create an expert library in Eclipse and create an Atlas in MIM. CT images of the other 20 patients were used to test the auto‐segmentation. In the Y‐90 procedures, liver contours were manually delineated by expert physicians and the manually delineated contours were used in Y‐90 activity calculations.

**TABLE 1:** Characteristics of the patients

	Range (average ± std)
Age	47–84 (62 ± 10)
Liver volume size (cm^3^)	1057–4284 (1997 ± 756)

In Eclipse, when the Smart Segmentation was initiated, the software calculated similarity between the test case and expert cases and provided a similarity ranking of the expert cases for a user to select an expert case for auto‐segmentation. After an expert case was selected by the user, image registration and contour deformation were carried out. In MIM, when the Atlas Segment was conducted, the software searched in the Atlas to find a subject, which had the best match with the test case, then performed image registration and deformed the contours of the subject to the test case.

In both of the applications, when the software detected that the automatic alignment between the test case and the subject or expert case was poor, the software asked the user to choose if the user wanted to continue with the automatic alignment or to conduct a manual alignment. In such cases, we performed a manual alignment.

To evaluate the auto‐segmentation results, Dice similarity coefficient (DSC) (Equation 1), mean distance to agreement (MDA), and ratio of volume (RV), between automatically segmented and manually delineated contours, were calculated. The manually delineated contours were taken as the standard.

(1) $$DSC\;=\frac{2\left|A\cap B\right|}{\left|A\right|+\left|B\right|}\;$$

where *A* and *B* are manually delineated and automatically segmented volumes, respectively. The DSC quantified the overlap between two contours: “1” represented a perfect overlap and “0” represented no overlap.

MDA represented the average distance between two contours (automatically segmented and manually delineated). The smaller the MDA, the better the contour agreement.

RV was the ratio of automatically segmented volume to manually delineated volume, which indicated the difference between these two volumes in the following:

(2) $$RV\;=\frac{\left|B\right|}{\left|A\right|}\;$$

The contours generated with Eclipse Smart Segmentation were imported into MIM for comparison. All the DSC, MDA, and contour volumes were calculated in MIM.

A further test was performed to assess Y‐90 activity calculations using the automatically segmented liver volumes. The following equation is the BSA method used for determining Y‐90 activity^2^:

(3) $$TA\;\left(GBq\right)=\;BSA-0.2+TI$$

where *TA* is the total activity, *BSA* is the activity determined with a patient height and weight, and *TI* is tumor involvement:

(4) $$TI\;=\frac{V_{T}}{V_{L}}\;$$

where *V_T_* and *V_L_* are tumor volume and liver volume, respectively. Because the test was focused on checking the effect of using the automatically segmented liver volumes in Y‐90 activity determination, the automatically segmented liver volumes were used for *V_L_* and the tumor volumes obtained from manual delineations were used for *V_T_* in the activity calculations.

Ratio of activity (RA), that is, ratio of the activity calculated using automatically segmented liver volume (*TA_auto_*) to the activity calculated using manually delineated liver volume (*TA_manual_*), which was the standard, was used to evaluate activity deviations from the accurate values. “1” Represented no deviation:

(5) $$RA\;=\frac{TA_{auto}}{TA_{manual}}\;$$

In the comparisons, the Wilcoxon signed rank test was conducted to test difference significance and a significance level of 0.05 was applied.

## 3 RESULTS

**FIGURE 1:**
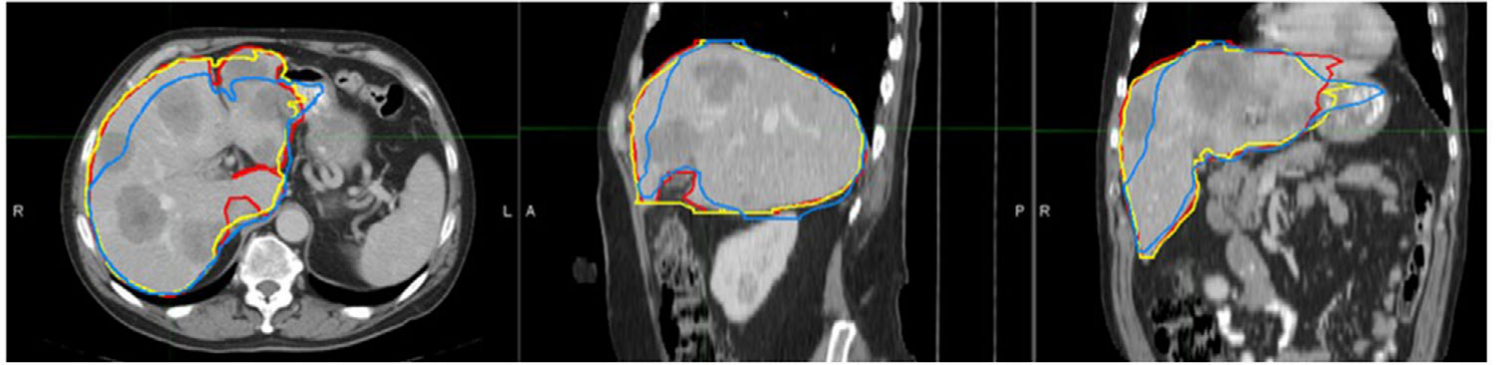
Liver contours generated by manual delineation (red), Eclipse Smart Segmentation (blue), and MIM Atlas Segment (yellow), shown in axial (left), sagittal (middle), and coronal (right) views

Figure 1 shows an example of liver contours generated with Eclipse Smart Segmentation, MIM Atlas Segment, and manual delineation, respectively.

**FIGURE 2:**
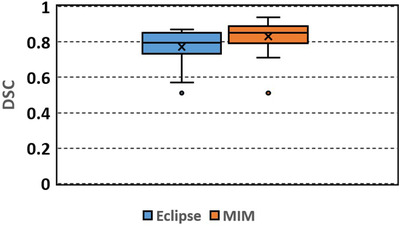
Box plot of Dice similarity coefficient (DSC) between the automatically segmented (Eclipse, MIM) and manually delineated liver contours

**FIGURE 3:**
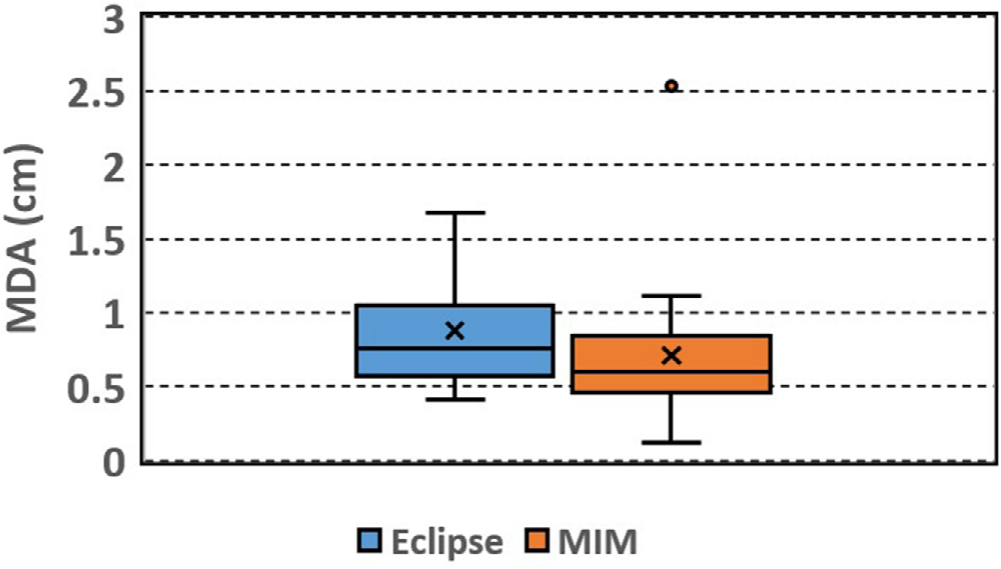
Box plot of mean distance to agreement (MDA) between the automatically segmented (Eclipse, MIM) and manually delineated liver contours

**FIGURE 4:**
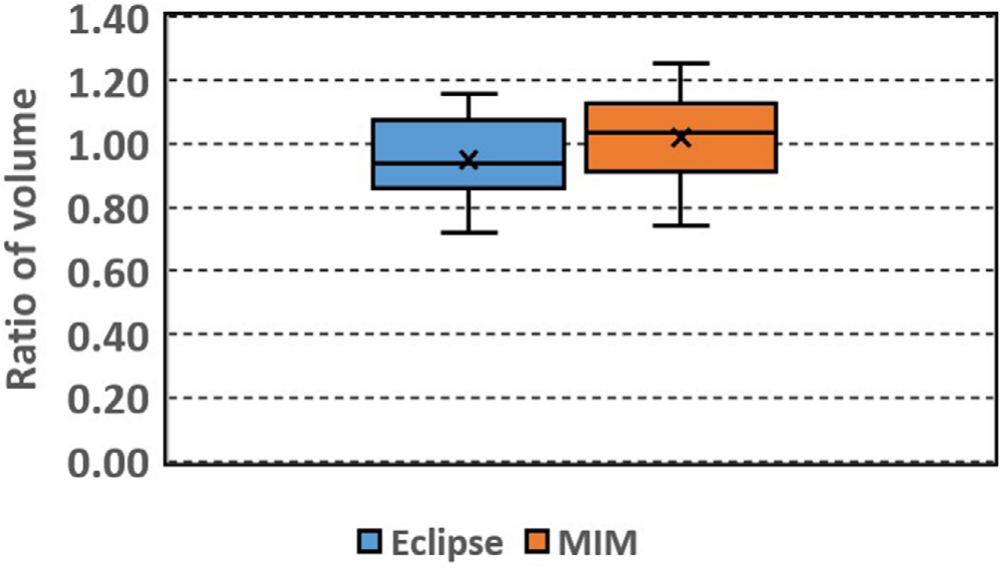
Box plot of ratio of volume (RV): ratio of the automatically segmented (Eclipse, MIM) to the manually delineated liver volume

**FIGURE 5:**
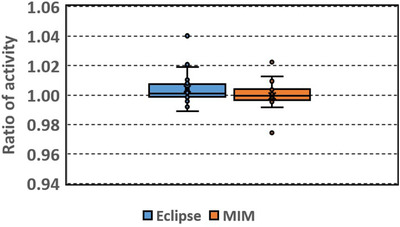
Box plot of ratio of activity (RA): ratio of the activity calculated using the automatically segmented (Eclipse, MIM) to the activity calculated using manually delineated liver volume

Figures 2, 3, 4, 5 show box plots of DSC, MDA, RV, and RA, respectively. Table 2 lists the mean and standard deviation. DSC ranges from 0.51 to 0.87 (mean 0.77, median 0.80) and 0.51 to 0.94 (mean 0.83, median 0.85) for the Eclipse and MIM results, respectively. The DSCs of the Eclipse and MIM results have outliers of 0.51. The poor performances of the auto‐segmentations might be attributed to the poor image contrasts of the livers in the images. In these cases, the livers have very similar image intensities as the adjacent tissues.

**TABLE 2:** Results of dice similarity coefficient (DSC), mean distance to agreement (MDA), ratio of volume (RV), and ratio of activity (RA) (*N* = 20)

	DSC	MDA (cm)	RV	RA
Eclipse	0.77 ± 0.1	0.88 ± 0.38	0.95 ± 0.13	1.00 ± 0.01
MIM	0.83 ± 0.09	0.71 ± 0.49	1.02 ± 0.14	1.00 ± 0.01
*p* Value	0.01	0.02	0.09	0.124

MDA ranges from 0.41 to 1.67 cm (mean 0.88, median 0.76 cm) and 0.12 to 2.53 cm (mean 0.71, median 0.60 cm) for the Eclipse and MIM results, respectively. The MDAs of the MIM results have an outlier of 2.53 cm, which occurs in the case where the DSC is the smallest. The outlier again indicates the poor performance of the auto‐segmentation in the case. The rest of the MDAs of the MIM results are within 1.11 cm.

RV ranges from 0.72 to 1.16 (mean 0.95, median 0.94) and 0.74 to 1.25 (mean 1.02, median 1.03) for the Eclipse and MIM results, respectively.

RA ranges from 0.99 to 1.04 (mean 1.00, median 1.00) and 0.97 to 1.02 (mean 1.00, median 1.00) for the Eclipse and MIM results, respectively. RAs have outliers of 1.02 and 1.04 for the Eclipse results, and 0.97 and 1.02 for the MIM results, respectively. The rest of the RAs are between 0.99–1.02 and 0.99–1.01 for the Eclipse results and MIM results, respectively. The wider distribution of RA of the Eclipse results implied larger activity deviations.

Among the automatically segmented contours generated with Eclipse, 50% of the contours had DSC over 0.8 and 75% of the contours had DSC over 0.74. Among the contours generated with MIM, 50% of the contours had DSC over 0.85 and 75% of the contours had DSC over 0.8. Overall the contours generated with MIM had slightly larger DSC (*p* = 0.01) and smaller MDA (*p* = 0.02) than those generated with Eclipse. The RV and RA did not show significant differences between the Eclipse and MIM results (*p* = 0.09 and 0.124, respectively).

## 4 DISCUSSION

In this study, both of the auto‐segmentation tools are Atlas based, and the Atlas in MIM and the expert library in Eclipse were built with the same CT image set. The results of DSC and MDA indicate that MIM Atlas Segment performed better than Eclipse Smart Segmentation.

In Eclipse, there is no optional setting for auto‐segmentation. In contrast, MIM provides a few options for users to select. In the study, we used the default setting, and the Majority Vote was used as the finalized method. The mean DSC of MIM results (0.83) was smaller than that (0.93) in La Macchia et al.’s study,^13^ where Atlas Segment of an earlier version of MIM (version 5.1.1) was used to generate liver contours in pleural cancer patients, and the atlas was built with five patients’ CT images. The smaller DSC in our study might be due to the quality of the CT images of livers. The patients in our study were liver cancer patients. Different image intensities of tumors and normal liver tissues within a liver might bring challenges to the auto‐segmentation to generate accurate liver contours in these cases. Casati et al.’s study on pelvis patients showed that optimized workflow and setting options in MIM can improve the auto‐segmentation.^6^ It is anticipated that the auto‐segmentation performance of MIM can be improved by using an optimized setting in our future study.

The results that RAs were close to 1, which showed that Y‐90 activities calculated using the liver volumes generated with these two auto‐segmentation tools were close to the accurate activities calculated using the manually delineated liver volumes. The maximum deviation from the accurate activities was 4%. The results indicate that both of these two commercial tools can be applied for liver delineation for Y‐90 SIRT procedures. The automatically segmented initial contours, with physician's slight editing, will be able to generate accurate activities. In our institution, a multidisciplinary team is involved in Y‐90 SIRT procedures: radiation oncologists contour the structures, medical physicists calculate Y‐90 activity using a patient's height and weight and the structures’ volumes, a lab prepares Y‐90 microsphere vials for a treatment following the activity calculation, and interventional radiologists deliver the treatment. The efficiency of the procedure workflow (from activity calculation to delivery) often relies on the activity calculation process, which relies on the contouring process. If auto‐segmentation can be successfully applied in SIRT, that is, auto‐segmented volumes can be used directly or after slight editing, the activity calculation process can be expedited and the efficiency of the workflow can be improved. Expedited activity calculations are important, especially in emergent cases, which need a quick turnaround from the activity calculation to the treatment.

In this study, CT images of 22 patients were used as the expert cases in Eclipse and as the Atlas subjects in MIM. Lee et al.’s study of Atlas‐based auto‐segmentation in head‐and‐neck patients showed that generally Atlas segmentation performance could be improved as the Atlas library was increased.^5^ It is anticipated that the auto‐segmentation performances of these tools for liver delineation can be improved if the expert library or the Atlas includes more expert cases or subjects.

## 5 CONCLUSIONS

MIM outperformed Eclipse in both DSC and MDA. The liver volumes and the resulted Y‐90 activities did not show significant differences between Eclipse and MIM results. Both auto‐segmentation tools can be used to generate initial liver contours for resin Y‐90 SIRT, which need to be reviewed and edited by physicians.

## CONFLICT OF INTEREST

The authors declare that there is no conflict of interest that could be perceived as prejudicing the impartiality of the research reported.

## AUTHOR CONTRIBUTION

Jun Li designed the study, analyzed the data, and wrote the manuscript. Rani Anne contoured the volumes and reviewed the manuscript.

## Data Availability

The data that support the findings of this study are available on request.
